# Retraction: ANRIL promotes chemoresistance via disturbing expression of ABCC1 by regulating the expression of Let-7a in colorectal cancer

**DOI:** 10.1042/BSR20180620_RET

**Published:** 2026-03-26

**Authors:** Pengfei Liu, Wei Duan, Zhen Zhang, Lifeng Feng

**Affiliations:** 1 The Department of Gastroenterology, Yan'an Univer­sity Affiliated Hospital, Yan'an, China; 2 The Department of Oncology, Yan'an University Affiliated Hospital, Yan'an, China

**Keywords:** Chemoresistance, Colorectal carcinoma, LncRNA, MiRNA

This article is being retracted from *Bioscience Reports* at the request of the Editor-in-Chief and the Editorial Board.

This follows the receipt of a notification from a reader, alerting the Editorial Board to unexpected similarities between the Figure 2G bottom si-NC colony assay, after a 90-degree rotation, and the Figure 5C OSCC3/si-NC colony assay from Zhang et al. 2017 (doi: 10.1038/s41598-017-13431-y). The authors were contacted with regards to the concerns and retraction but have not responded to the Journal's queries or the concerns raised.

Given the extent of the issues raised, the Editorial Board stand by the decision to retract the article.

